# Changing sexual behaviours amongst MSM during the COVID-19 restrictions in Wales: a mixed methods study

**DOI:** 10.1186/s12889-022-12821-w

**Published:** 2022-02-25

**Authors:** Adam Williams, David Gillespie, Zoë Couzens, Fiona Wood, Kathryn Hughes, Kerenza Hood

**Affiliations:** 1 Centre for Trials Research, School of Medicine, College of Biomedical & Life Sciences, Cardiff University, Wales, UK; 2 Public Health Wales NHS Trust, Wales, UK; 3 PRIME Centre Wales, Division of Population Medicine, School of Medicine, College of Biomedical & Life Sciences, Cardiff University, Wales, UK

**Keywords:** Sexual health, Sexual and gender minorities, COVID-19, Behaviour Change, Mixed methods

## Abstract

**Background:**

The COVID-19 pandemic and its associated restrictions stopped people freely engaging in sexual behaviour. We explored sexual behaviour amongst men who have sex with men (MSM) using mixed methods during the multiple lockdowns in Wales.

**Methods:**

An online survey was advertised on social media platforms (focusing on Welsh LGBT groups), from June 2020 to July 2020. MSM over 16 years were invited to take part if they were resident in Wales. Qualitative interviews were undertaken as part of a study examining knowledge and awareness of sexual health. Interviews were conducted between September 2020 and February 2021 via Zoom©. Interview data was analysed thematically and integrated with survey data.

**Results:**

The survey received 70 responses, 60% (*n *= 42) reported not having sexual activity during lockdown. Restrictions altered the number of new sexual partners per week with over 80% (*n *= 56) not having any new sexual partners for the 12 weeks of the first lockdown. However, as the weeks progressed following the first lockdown there was an increase in the number of new sexual partners. Interview data indicated that the COVID-19 pandemic had a large impact on reducing sexual behaviour with other MSM in Wales. ‘Lockdown fatigue’ was viewed to result in different levels of adherence to the lockdown rules depending on the lockdown being discussed. Of those engaging in sex outside the rules, ‘shame’ was commonly reported. The restrictions were believed to have a positive impact on reducing the spread of sexually transmitted infections.

**Conclusions:**

The COVID-19 pandemic and associated restrictions had a significant impact on sexual behaviours among MSM in Wales, with the majority fully adhering to the lockdown rules. Although the population were largely compliant with the lockdown restrictions, lockdown fatigue may suggest that any future lockdowns might not have the same effect.

**Supplementary Information:**

The online version contains supplementary material available at 10.1186/s12889-022-12821-w.

## Background

The COVID-19 pandemic has resulted in unprecedented lifestyle restrictions globally. The pandemic resulted in the UK government imposing a ‘lockdown’ on 23 March 2020 with gradual easing from June 2020. However, from this point the four nations of the UK started to diverge in terms of their approach to COVID-19 restrictions. In Wales, restrictions continued to ease until September 2020 when local lockdowns started to occur in response to the rise in infections. The continued rise then resulted in a 17-day ‘circuit break’ in Wales (23rd October to 9th November) with new Wales specific measures imposed following that. The Welsh Government intended to allow ‘bubbles’ to form for 5 days over the Christmas period. However, on the 19th December the Welsh Government announced an emergency move into Alert level 4 with the Christmas bubble only applicable to the 25th December. On 4th January 2021, Wales returned to alert level 4 (highest restrictions in place, only allowed to meet those in support bubble, business closures and travel restrictions), entering its third lockdown until 12th March 2021 [1].

During these periods of lockdown, members of the public were forced to stay at home and only permitted to leave under certain circumstances. Meeting individuals outside of one’s household was not permitted, with police enforcing restrictions. Outside of the lockdown periods, restrictions on meeting individuals indoors were limited to those who formed their extended household and people were required to avoid entering other households.

One area majorly impacted by social restrictions was engagement in sexual activity. British Association for Sexual Health and HIV provided guidelines to members of the public that discouraged sexual contact with anyone outside the household due to the potential for transmission [2]. In response to GUM clinic closures, Wales provided a free postal testing service for HIV and STIs which was active from May 2020 [3]. The few studies that have explored the adherence to COVID-19 restrictions among men who have sex with men (MSM) have found varying levels of adherence. One study conducted by a London Sexual Health Clinic identified that half of participants continued to be sexually active [4]. In contrast, research from studies in South Wales and Australia found a higher adherence to restrictions among MSM over a similar period [5, 6]. In this study we aimed to explore sexual behaviour amongst MSM in more depth by triangulating results from an online survey and qualitative interviews during a period of lockdowns in Wales.

## Materials and methods

### Design

Retrospective online survey and qualitative interviews conducted via Zoom©.

### Participants

All participants self-identified as MSM currently living in Wales and aged 18 years and over. 

## Data collection

### Survey

A self-completed online survey was developed using Qualtrics©. Piloted among a small sample of MSM. The survey included questions on socio-demographic characteristics, risk and sexual health testing, sexual partners and sexual contacts, views on COVID-19, sexual activity during lockdown and access to sexual health products/services. Survey provided in Supplemental Material.

A web link to the survey was posted on various social media platforms including Facebook (targeting LGBT+ groups), Grindr and Twitter. All questions within the survey related to the first UK lockdown ranging from 23rd March 2020 to the 27th June 2020. The survey was live from June 2020 to July 2020.

### Interviews

 Qualitative interviews were undertaken as part of a larger study which explored participants’ views of the relationship between HIV pre-exposure prophylaxis (PrEP), sexually transmitted infections, and antibiotic resistance. The open-ended topic guide for this study included questions exploring the impact of COVID-19 on sexual behaviour. The data for this paper were obtained from this section of the interview (see Interview Transcript in Supplemental Material). Interview participants were recruited through response to advertisement (same as described for the online survey) and snowballed from initial participants.

All interviews were conducted by AW and lasted between 30 and 60 min. Interviews were conducted between September 2020 and February 2021 using remote methods of data collection (Zoom©). Informed consent was obtained prior to data collection and verbal consent audio-recorded separately. Participants were gifted a £20 voucher for their time. The sample size was informed by ‘information power’ [7]. The narrow aims of the interviews, participants belonging to specified target groups and strong dialogue between participants and interviewer led the researcher team to conclude that the sample size was sufficient at twenty participants.

## Analysis

### Survey

Descriptive analyses were performed on the survey responses using IBM SPSS Statistics 26. Participants who did not complete all questions were excluded from the analysis.

### Interview

Interviews were digitally audio-recorded, transcribed verbatim by a professional transcriber, anonymised, imported into NVivo 12 (QSR International) and analysed thematically by AW [8]. A subset of transcripts were independently analysed by FW and DG to aid code refinement and maximise rigour. Codes were then discussed amongst these three analysts and agreed. Additionally, 10% of transcripts were double coded by FW using the finalised coding framework to review coding reliability.

## Triangulation

Within this study, a triangulation protocol method was adopted. There are three main approaches used for triangulation, but this was deemed most appropriate as it allows for datasets to be analysed separately. Triangulation occurs after analysis and combines the findings from both datasets to form the interpretation of findings [9]. Due to data collection occurring at different times the triangulation protocol approach fitted the circumstances better.

## Results

### Survey findings

Overall, 70 MSM responded to the online survey. The median age of participants was 33 years (IQR 18-66). The sample was largely white (n=66, 94%), educated with a university degree or higher (*n *= 45, 64%) and single (*n *= 43, 61%), full results detailed in Supplemental Material. Of these, 60% (*n *= 42) reported no sexual activity during lockdown with the main reason being that they were trying to limit social contact (*n *= 28, 67%). Other reasons included no privacy where they lived (*n *= 5, 18%) and not wanting sex (*n *= 4, 14%). Of those sexually active during lockdown (outside the household), 83% (*n *= 23) engaged in sex with someone outside of their household during lockdown. Most participants reported that they had fewer sexual partners during the lockdown than prior to lockdown (*n *= 52, 74%). Half of participants were concerned about contracting COVID-19 (*n *= 33, 51%), and 89% (*n *= 62) agreed with the lockdown measures.

**Fig. 1:**
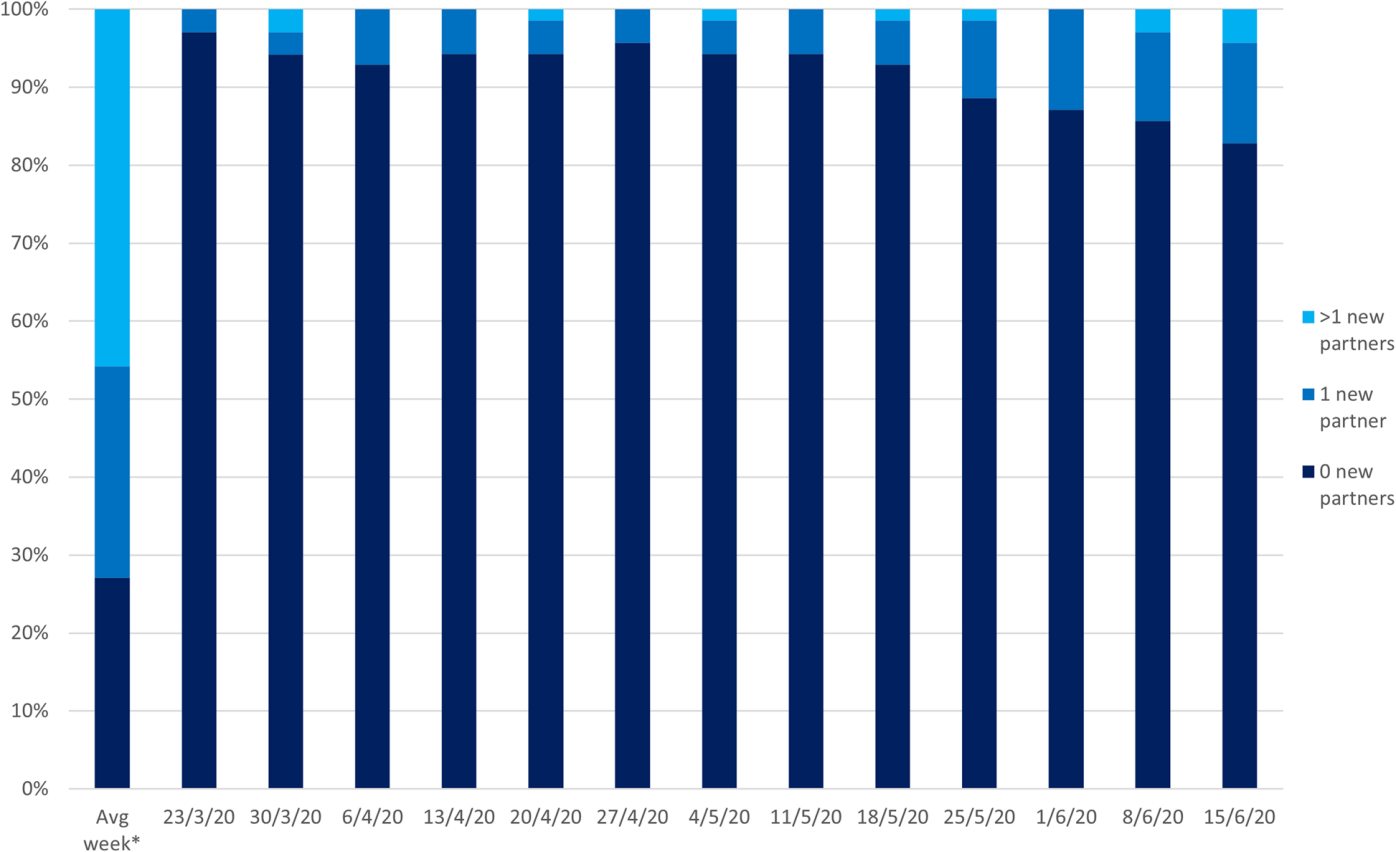
Number of new sexual partners in the first 12 weeks of lockdown. A graph visually representing the survey data relating to the number of new sexual partners reported each week of the lockdown commencing the week starting 23/03/2020 until week commencing 15/06/2020. With an average week pre-COVID included as a comparator. Shows an increase in the number of new sexual partners as weeks progress through lockdown. (Created by the authors)

Figure 1 presents how the lockdown measures reduced sexual behaviour of participants with almost half (*n *= 32, 46%) reporting that in an average week (pre-COVID) they would engage in sexual activity with two or more new partners. In comparison, during the 12 weeks of lockdown (23rd March 2020 to 15th June) over 80% (*n *= 56) reported no new sexual partners each week. However, the survey data also indicated that the number of participants engaging in sex with new partners subsequently increased as each week passed from 23rd March.

**Fig. 2:**
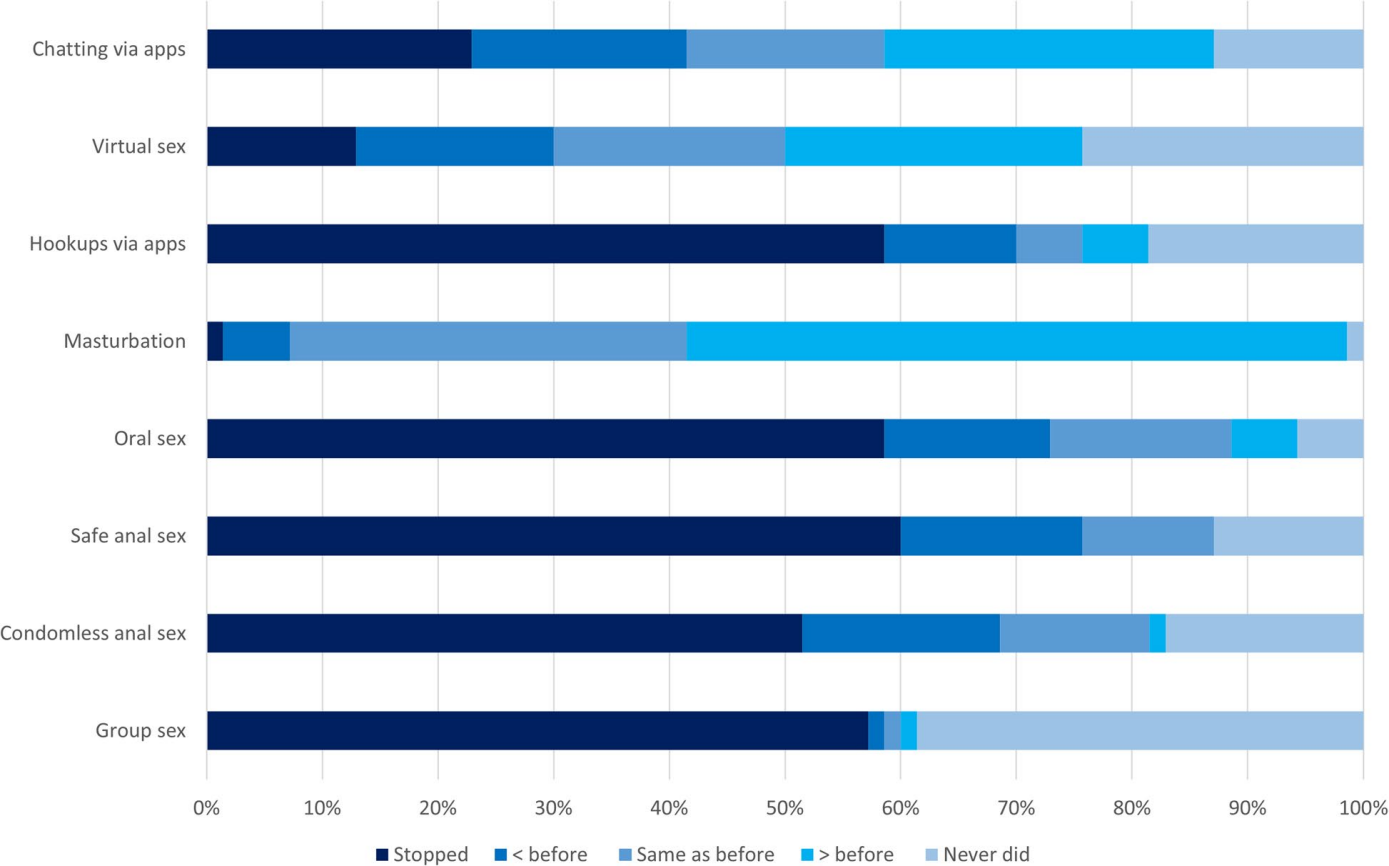
Reported behaviour changes resulting fromCOVID-19 lockdown measure*s.* A graph showing changes across various sexual behaviours resulting from the lockdown measures. Participants were asked to consider how behaviours changed from a pre-COVID point to their current situation (under full lockdown measures). (Created by the authors)

Findings also indicated that many sexual behaviours were reduced or stopped: (see Fig. 2) 70% (*n *= 49) stopped or reduced engaging in ‘hook-ups’ via apps (using apps to meet people for sex), 73% (*n *= 51) stopped or reduced oral sex, anal sex (with and without a condom) and group sex were stopped or reduced (*n *= 53 76%, *n *= 48 69% and *n *= 40 57%) respectively. Perhaps in response to this reduction in sexual behaviour with others, masturbation was reported to increase among many participants (*n *= 39, 56%). Virtual sex showed a mixture of responses across all options.

### Interview findings

Twenty participants were interviewed, ages ranging from 19 to 53 years. Most participants reported being white British, gay (both 85%) and single (70%).

### Overall impact on sex

Interview data indicated that the Covid-19 pandemic and lockdown measures enforced had resulted in major changes to participants’ own and others’ sexual behaviour. It was commonly reported that they themselves and others adhered stringently to the lockdown rules and avoided engaging in sexual contact with anyone outside of their household. Although, as with most rules, it was acknowledged that there were some who would not follow the rules and continue with their behaviour regardless of the pandemic.

*“There has been definitely a shift in people’s opinions on going out and getting sex in a number of people that I speak to and that I know personally. However, I also know that throughout the lockdown, some people were still engaging in like sex parties or very, very risky, unhealthy behaviours.” P01*.

However, as lockdowns started to ease it was believed that people took this as a green light to start to engage in sexual activity with others. Despite social distancing measures and rules against entering one another’s homes being in place.

*“Well, I think last year it [first restrictions] definitely did [reduce sexual activity], but I don’t think it’s going to be long lasting. I think it’s going to… my perspective of it is the sooner lockdown’s… well, the moment lockdowns start to ease, people are going to be, in some ways, a bit worse than they were before, because they’re going to have missed that social contact. After the main lockdown of March and May last year, my friends were definitely a lot more wanting to do stuff because they thought, oh well, we can now. I don’t think we’re going to find people have less sex or do less things. But I think they’re just more restrained while we have lockdowns in place. The moment they get lifted, most of my friends are straight back onto Grindr.” P19*.

One participant who had not engaged in sexual activity throughout the pandemic indicated that when restrictions are removed it would lead to the need for an enhanced conversation relating to sexual activity with future partners. His statement suggested that the pandemic may have enhanced his awareness and concerns relating to the transmission of infectious diseases, beyond solely STIs.

*“I, during COVID, have physically touched no one and I intend to continue that way. But that’s a personal choice. But I think, you know, anything would have to… emerging from COVID, whenever that happens, involve probably an even deeper conversation than would have been the case previously.” P04*

### Lockdown fatigue

Interview data indicated that some participants viewed there to be different levels of adherence to the lockdown rules depending on the lockdown being discussed. The initial lockdown from March was seen to be most widely accepted with the majority following the rules and not meeting people outside their household for sex. As mentioned previously, the first easing of lockdown in July 2020 was viewed by some as an approval to return to normal in terms of sexual behaviour. The circuit break and winter lockdown were perceived to have had less impact on people’s behaviours as people were more likely to continue meeting people for sex.

*“At the start of lockdown, the first one in March, I feel like it was just like the general consensus that most people weren’t meeting. But we’re in a full lockdown now [winter lockdown] and I don’t think people have the same attitude.” P16*.

Although, the seriousness of the situation that led to the winter lockdown and increasing restrictions throughout latter months of 2020 did lead some to alter their behaviours and adhere to social distancing rules despite breaching them throughout the summer and autumn months.

“I went through a period between the initial lockdown and July where I did not have sex at all. The sod it button came out and I thought, oh f*** it, I’ll just go and… so that’s when I started being able to take PrEP. And was a little more active between about July and November. It tended to be people I already knew, people I’d maybe met with a dozen times or so before, regular partners but on a casual basis. I’ve not done anything since December, as things [Covid-19 cases] have got worse, progressively.” P13.

### Shame

Among those who reported engaging in sexual activity during the lockdown, some described a sense of shame around the activity. They also described how sexual intercourse and intimacy was something they needed at that time to cope with the difficulties arising from the pandemic and restrictions. Suggesting sex is an activity to ease mental distress and ‘escape’ reality.

*“I would be telling a lie if I said that I hadn’t hooked up with somebody during this… during this pandemic. Does that make me proud? Does that make me happy? No. But is it what I’ve needed in that moment to… you know, to not feel completely shut off from everything? And also, just to kind of… also sex is escapism, to forget this massive, horrible situation that is happening right now, right? And again, I’m not here to moralise anybody.” P05*.

This sense of shame around having sex during the pandemic was mentioned by many participants with conversations suggesting that people have become “fearful about saying that they’ve had sex with somebody, because they’re going to be judged because of the whole Covid thing” (P02). This is believed to have led to sex being more hidden with people not engaging with sexual health services due to them not wanting to admit that they are having sex during the lockdowns.

*“They aren’t going to a sexual health clinic because they’re not supposed to be out and about. When the whole Covid… when the whole lockdown, there was people from the group that were still going out, having unprotected sex and having sex with people. And it was only afterwards when we got to the end that we found out that some… that some people were [having sex]. So, it’s kind of… I think what it’s done is kind of driven it a bit more underground.” P02*.

### Impact of restrictions

Despite the pandemic and associated restrictions having a negative impact on people’s sex lives, some participants were able to identify some positive aspects. For example, some believed that the restrictions on social activities had resulted in a reduction of sexually transmitted infections. As one participant asserted: *“This is really a firebreak, an STI firebreak.” (P08)*.

*“I think culturally COVID-19 has forced people to limit the amount of people they see and limit the amount of like random encounters that people have. So, in terms of spreading STIs and STDs, it’s limited in that sense, because people are meeting less people.” P10*.

However, concern was raised that despite there being a reduction in spread, there may also be a reduction in testing. Although in Wales postal testing for STIs was introduced while GUM clinics were closed, there was a concern that many people were not aware of the postal testing service. This could lead to an increase in spread if people are continuing to meet for sex but not getting tested/treated.

*“But general sleeping around has got to have reduced over that time. If there was proper, you know, testing like these postal tests, like I did, around that time, I would imagine it’s had a positive effect in reducing the amount of STIs spreading around. But on the other hand, if people don’t know about those tests… the postal tests, like you said, they might have, you know, not… they might have missed two or three opportunities to go to a clinic when they had something.” P03*.

Alternatively, while the lockdown and restrictions are seen to have a positive impact on the transmission of STIs, many of those interviewed believe that once all restrictions are lifted there will likely be a large rise in STI rates due to a surge in sexual activity among MSM.

*“I say a bit of a spike, a huge orgy of activity once lockdown is finally lifted. And… yeah. I think everyone’s going to go wild, quite frankly. So that’ll be interesting to see how that happens.” P13*.

## Discussion

Our survey findings suggest that during the first lockdown there was a high level of adherence to the restrictions among the study population, with many MSM avoiding having sex with others outside of the household. Behaviours were reported to have changed dramatically in relation to reducing sexual activity with others. Solo masturbation may have increased to account for the reduction in sex with others, but virtual sex has not been as popularly adopted as some might expect. Our survey data indicate that as July started there was an increase in the number of new sexual partners. This is supported by our qualitative data with participants suggesting that the easing of restrictions was seen by some to mean they could return to having sex with those outside of their household/bubble. The data indicates that fewer people adhered to any subsequent lockdowns as well as the first. However, the conditions that led to the winter lockdown, the large increases in Covid-19 infections, hospitalisations, and deaths, may have had a greater impact on changing behaviour than the circuit break as suggested from the interviews.

Across both studies, most participants reported not engaging in sexual activity during the initial lockdown between March and July. These findings are similar to the Welsh cohort study [5] and online survey study in Australia among MSM [6]. Interestingly, far higher rates of sexually activity were identified from an online survey study exploring COVID-19 restrictions and changing sexual behaviours among MSM in London [4].

Those who reported breaking social distancing restrictions to have sex explained how at the time it was something that they needed in order to cope with the isolation/loneliness experienced since the pandemic began. However, many also felt ashamed of their behaviours and having to balance their own needs with what is best for the community/society. The reasons for engaging in sexual activity and guilt experienced are akin to findings identified in the London online survey study [4].

Changes in behaviour to the extent reported will likely result in a break in the transmission of HIV/STIs among MSM, which may lead to a reduction in these infections. The impact of the change in behaviour will be more profound if the previously sexually active MSM continued to be tested and treated for any underlying infections. While among the interview sample there was a high level of awareness of postal testing in Wales, only half reported awareness of its existence in the online survey. However, it had only recently been launched when the online survey was conducted. The COVID-19 restrictions and associated lockdown have provided a unique opportunity to reduce the previously accelerating STI rates identified in Wales but also across the world [10, 11]. However, this will require funding and focus to be placed on sexual health by countries health agencies, as rates of infections such as gonorrhoea and chlamydia could quickly return to pre-pandemic levels if there is a return to previous levels of sexual activity.

### Strengths and limitations

A key strength of this study is the use of mixed methods data analysis. It allowed us to explore the complexities and changes around sexual behaviour in a more detailed manner. The timing of this research allowed behavioural data to be collected during the ongoing situation rather than relying on memories of previous behaviour, providing additional benefits. Although some recall bias may still be affecting the responses.

The survey had a small sample despite its wide reaching and repetitive promotion. A major difficulty was getting to get related groups/services to promote the online survey. Many provided no response to enquiries, which may have been due to the difficulties the third sector organisations were experiencing during the initial lockdown.

The small sample size may have resulted in self-selection biases with potential impact on the results being unknown. Both the survey and interviews were not ethnically diverse but matched typical MSM research in terms of demographics of the populations (white, middle-aged, well-educated gay cisgender men) [4–6]. Future research is needed to explore how to better engage alternative MSM groups with sexual health research.

## Conclusions

Our findings suggest that there has been a high level of adherence to the lockdown rules and restrictions by MSM in Wales. However, adherence has likely waned as time has passed since the initiation of the restrictions. With the initial lockdown having the greatest impact on sexual behaviour with subsequent lockdowns being less effective. There is a continued need for the provision of sexual health services not only for test and treat, but also for sexual wellbeing to address the potential impact of shame that may be growing. Improvements in the promotion of sexual healthcare are required to enhance the impact of restrictions breaking transmission of STIs.

## Supplementary Information

**Additional file 1: **Supplementary Material

## Data Availability

The anonymised datasets generated and/or analysed during the current study are available at the discretion of the lead author, Adam Williams.
