# Supplementary material for: Changing sexual behaviours amongst MSM during the COVID-19 restrictions in Wales: a mixed methods study
Source: BMC Public Health. 2022 Feb 25;22:396. doi: 10.1186/s12889-022-12821-w (PMC8874301; doi:10.1186/s12889-022-12821-w)
Supplement: Supplementary file 1 — Additional file 1: Supplementary Material [file 12889_2022_12821_MOESM1_ESM.pdf]

Supplementary Material

1. Online Survey

CONSENT TO PARTICIPATE To take part in this study you must fit the criteria below: · **I am 18 years or older** · **I am a man who has sex with other men** · **I currently live in Wales** · **I have had sex with another man in the last 12 months** Do ALL of the listed items describe yourself?

Yes (1) No (2)

Skip To: End of Survey If CONSENT TO PARTICIPATE To take part in this study you must fit the criteria below: · I a... = No

I agree to participate and for my answers to be used in this survey.

Yes (1) No (2)

Skip To: End of Survey If I agree to participate and for my answers to be used in this survey. = No

End of Block: Information and Consent

Start of Block: Section A) About You

How old are you? ____

How would you describe your ethnicity?

▼ White (1) ... Prefer not to say (7)

What region of Wales do you live?

▼ Bridgend (1) ... Wrexham (21)

What is your highest qualification?

▼ I have no educational qualifications (1) ... Other (please state) (6)

What is your relationship status?

▼ Single (1) ... Other (9)

How would you best describe your gender?

Man (1) Non-Binary (2) Trans (3) Other (4)

Which best describes your sexuality?

Gay(1) Bicurious(2) Bisexual(3) Straight(4) Pansexual(5) Queer(6) Other(7)

In the last 12 months, whom have you had sex with? (Tick all that apply)

Men (1) Trans men (2) Trans women (3) Women (4) Genderqueer/Non-Binary individuals (5)

What is your HIV status?

HIV negative and tested in last year (1) HIV Negative but tested longer than a year ago (2)

HIV Positive (3) Unknown (4) Prefer not to say (5)

End of Block: Section A) About You

Start of Block: Section B) Risk and Sexual Health Testing

Display This Question:

If What is your HIV status? = HIV negative and tested in last year

And What is your HIV status? = HIV Negative but tested longer than a year ago

And What is your HIV status? = Unknown

And What is your HIV status? = Prefer not to say

Are you currently taking PrEP (daily or event based) or PEP?

Yes PrEP (1) Yes PEP (2) No (3) No, being treated for HIV (4)

Have you been diagnosed with HIV or STIs in the last 12 months?

No (1) or yes (2) option for Chlamydia (1), Gonorrhoea (2), Syphilis (3), Hepatitis C (4), HIV (5), Other (6)

In the last 12 months, have had sex with multiple people at the same time? E.g. threesome, gangbang, sex party etc Yes (1) No (2)

In the last 12 months, have you had sex whilst using a drugs (e.g. mephedrone, crystal meth etc.) or alcohol? Both drugs and alcohol (1) Just drugs (2) Just alcohol (3) Neither (4)

Which best describes the main reason for your last visit to a sexual health clinic?

Regular check up (1) I had symptoms (2) I had been at risk of HIV (3)

A sexual partner told me they had an infection (4) I had a new sexual partner (5)

For a PrEP Review (6) Other (Please Specify) (7)____

When was your last STI test? In the last month (1) 2 to 3 months ago (2) 4 to 12 months ago (3) More than a year ago (4) I have never previously tested for STIs (5)

Skip To: Q25 If When was your last STI test? = I have never previously tested for STIs

---

How often do you get a check-up for STIs, assuming you have no symptoms?

Every month (1) Every 3 months (2) Every 6 months (3) Once a year (4) Less than once a year (5)

If there was a time when you were more at risk of STIs (e.g. when you had more sexual partners), would this change how often you would be tested?

Yes (1) No (2)

Has the lockdown stopped you from getting tested? Yes (If yes, why?) (1)________________________ No (2)

Are you aware that Wales recently started STI/HIV at-home testing?

Yes (1) No (2)

End of Block: Section B) Risk and Sexual Health Testing

Start of Block: Section C) Sexual Partners and Behaviour

Sexual partners fall into 2 categories: Regular sexual partners – a person you are in a relationship with e.g. boyfriend/husband *or* a person you have repeated sex with but are not in a relationship e.g. friends

with benefits. Casual sexual partners – such as hook-ups, one-night stands, people met at saunas etc. How many sexual partners would you have in an average month? (Before Coronavirus and lockdown)

_______ regular partners (1)
_______ Casual partners (2)

What is your sexual position with sexual partners?

	Top [insertive] (1)	Bottom [receptive] (2)	Versatile [both] (3)
Regular partner (1)	○	○	○
Casual partner (2)	○	○	○

What percentage of the time will you use condoms with your sexual partners?

	0 10 20 30 40 50 60 70 80 90 100
Regular partners ()	
Casual partners ()	

Do you know the HIV status of your sexual partners before having sex?

	Always (9)	Most of the time (10)	About half the time (11)	Sometimes (12)	Never (13)
Regular partners (1)	○	○	○	○	○
Casual partners (2)	○	○	○	○	○

Where do you usually meet CASUAL partners? (Tick all that apply)

Friends (1) Grindr (2) Apps (not Grindr) (3) Bars (4) Sex on premises venues (saunas) (5) Cruising (6) Other (where?) (7) ____

End of Block: Section C) Sexual Partners and Behaviour

Start of Block: Section D) Sexual Behaviour during Lockdown

We would like to discuss your sexual activity during lockdown. Please remember this is **fully anonymous** so please answer honestly. The lockdown measures started on March 23rd 2020. For the purpose of this survey, we define sex activity as any activity between two or more people that induces sexual arousal, including masturbation and all oral and anal activities. This does not include virtual sex. Have you had sex during lockdown?

Yes (1) No (2)

Skip To: Q33 If We would like to discuss your sexual activity during lockdown. Please remember this is fully anon... = No

Whom are you having sex with?

regular partner/s I knew before the lockdown (1) regular partner/s I met during the lockdown (2)

casual partner/s I knew before the lockdown (3) casual partner/s I met during the lockdown (4)

Display This Question:

If We would like to discuss your sexual activity during lockdown. Please remember this is fully anon... = Yes

Do you live with the sexual partners you have had during lockdown?

Yes, all of them (1) Some of them (2) No, none of them (3) We moved in together for the lockdown(4)

Other [free text] (5)

Below is a list of each week of lockdown. Please indicate the number of causal sexual partners you have had each week. If you met the same person multiple times please only count them once (Put zero for none).

23rd March – 29th March (1) ___ 30th March - 5th April (2) ____ 6th April - 12th April (3) ___

13th April - 19th April (4) ___ 20th April - 26th April (5) ___ 27th April - 3rd May (6) ___

4th May - 10th May (7) ___ 11th May - 17th May (8) ___ 18th May - 24th May (9) ___

25th May - 31st May (10) ___ 1st June - 7th June (11) ___ 8th June - 14th June (12) ___

15th June - 21st June (13) ___

Display This Question:

If We would like to discuss your sexual activity during lockdown. Please remember this is fully anon... = No

What are your reasons for not having sex?

No privacy at where I live (1) No one wants to meet (2) Don't want sex (3) Trying to limit social interaction (4) Self-isolating due to showing symptoms/someone in household has symptoms (5) Other (6)

____

Have the number of sexual partners you have per week/month changed due to lockdown?
No, same number of partners as before lockdown (1) Yes, less partners now than before lockdown(2) Yes, more partners than before lockdown (3)

How have your behaviours on the following changed because of the Coronavirus lockdown? Answered: Stopped completely (1) Less than before (2) Same as before (3) More than before (4) Started since lockdown (5) Didn't do before or now (6)

Chatting via dating apps/ websites (1) Hookup via dating apps/ websites (2) Virtual sex (including sharing photo and video) (3) Oral sex (4) Masturbation (5) Mutual masturbation (6) Penetrative sex with condoms (7) Penetrative sex without condoms (8) Group sex (9) Using PrEP (10) Sex with toys (11) Sharing sex toys (12) Sex with payment (Receiving or giving) (13) Chemsex (e.g. use of illicit substances during sex) (14) BDSM (e.g. bondage, dominance and submission) (15) Public sex with casual partner(s) (e.g. cruising, dogging) (16)

Please explain some of the reasons why these behaviours have changed?

____

End of Block: Section D) Sexual Behaviour during Lockdown

Start of Block: Section E) Concerns about Coronavirus

Have you received a letter about shielding from the Government due to being part of the high-risk group? Yes (1) No (2)

Are you, or have you previously, self-isolating because you or someone in your household have symptoms of Coronavirus? *Yes (1) No (2)*

Are you a key worker? *Yes (1) No (2)*

Have you experienced any changes to physical or mental health due to the lockdown measures?

Yes (please provide a brief explanation) (1) ________________ *No (2)*

How concerned are you with catching Coronavirus? E*xtremely concerned (1) Somewhat concerned (2) Not concerned (3) Don't think about it (4)*

What do you think is your level of risk of catching Coronavirus? *Extremely likely (1) Somewhat likely (2) Neither likely nor unlikely (3) Somewhat unlikely (4) Extremely unlikely (5)*

Do you agree with the lockdown measures used? *Strongly agree (1) Somewhat agree (2) Neither agree nor disagree (3) Somewhat disagree (4) Strongly disagree (5)*

Do you wear a face covering when leaving the house? *Yes (1) No (2)*

Prior to the lockdown (23rd March), did you alter any of your behaviours (e.g. start to limit social interactions) when you heard Coronavirus cases were rising in Wales? *Yes (1) No (2)*

Has the Coronavirus made you more concerned about other contagious diseases/infections? *Yes (1) Somewhat (2) No (3)*

Skip To: End of Block If Has the Coronavirus made you more concerned about other contagious diseases/infections? = No

What infections do you now have higher concern for? (please tick all that apply)Flu/Common cold (1) HIV (2) Pneumonia (3) Severe acute respiratory syndrome (SARS) (4) Sexually transmitted infections (STIs) (5) Other (please state) 6) ______________________________

End of Block: Section E) Concerns about Coronavirus

---

2. Interview Schedule

UPrEP Qualitative Interviews: Interview Schedule

(START AUDIO RECORDER)

Please confirm your consent to the study and to be audio recorded by saying your name and the date. This interview is being conducted by Adam Williams with (participant ID) on (date) commencing (time).

First, I would like to discuss PrEP.

- How would you describe what PrEP is to someone?
- How did you first hear about PrEP?
- What encouraged your decision to start taking/not take PrEP?
    - [For those who have stopped PrEP]
    - Why did you stop taking PrEP? Will you start again?
    - Have you ever stopped taking prep? Why?
- What benefits do you believe there are to PrEP for you and others?
- Can you think of any issues/problems with PrEP?

Some people have suggested that PrEP use could reduce condom use.

- What do you think of their suggestion?
    - Query their response with why?
    - Do you think your condom use has changed since starting PrEP?
- In your opinion, do you think condoms are important?

- From your experience, do you think gay and bisexual men are concerned with protection from sexually transmitted infections?
- Would you say there are differing levels of concern?

Now if we could discuss sexually transmitted infections.

- How well do you think your knowledge is?
- Can you tell me a bit about some of the STIs you know about and your knowledge of how they can be passed on?
- Where do you get your knowledge from? Proactively looking?
- When entering into, or considering, a sexual encounter with someone, what safety measures do you typically think about?
- Are you concerned about catching an STI?
    - What do you think informs your level of concern?
- What impact, if any, do you think PrEP may have on STIs?
- What do you think the rate of STIs in gay and bisexual men is?
- Can you think of any ways to reduce STI rates among gay and bisexual men?
    - Easier/quicker access to testing?
    - More information about STIs?
    - More information about benefits of condoms?
    - More information about rates of STI infections in the community?

Moving to the next topic.

- Are you aware of the term antimicrobial or antibiotic resistance?
    - [If yes] could you explain where you have heard about this or what in relation to?
    - could you explain your understanding of it?

It is serious concern and most countries are implementing procedures to slow the development of resistance. One bacterial infection that has become increasingly resistant to multiple antibiotics is gonorrhoea.

- Are you concerned about AR STIs?
- Do you think there is much awareness of antibiotic resistant STIs?
- Do you think being made aware of antibiotic resistant STIs and its consequences would alter sexual practices among gay and bisexual men?
- What do you think would be the best way to inform gay and bisexual men about antibiotic resistant sexual infections?

Lastly, do you think Covid-19 will have any long lasting impacts on sexual behaviour or concerns around STIs?

- Is there anything else you think I should know or want to add?
- That concludes out interview, thank you for your time.

(STOP AUDIO RECORDER)

---

3. Overall results

Table 1. Overall results of the online survey displayed as total and according to Risk Behaviours reported.

	Total sample (n=70)	Higher risk (n=34)	Lower risk (n=36)
Median years (IQR)	33 (18-66)	34 (21-66)	31 (18-63)
White ethnicity, n (%)	66 (94.3)	33 (97.1)	33 (91.7)
Single, n (%)	43 (61.4)	24 (70.6)	19 (52.8)
University degree or higher, n (%)	45 (64.3)	21 (61.8)	24 (66.7)
Reported PrEP use, n (%)	17 (24.3)	12 (35.3)	5 (13.9)
Median percentage of condom usage with partners:			
Casual (IQR)	72 (0-100)	68 (0-100)	76 (0-100)
Regular (IQR)	38 (0-100)	26 (0-100)	48 (0-100)
Always know the HIV status of partners:			
Casual, n (%)	25 (35.7)	10 (29.4)	15 (41.7)
Regular, n (%)	47 (67.1)	22 (64.7)	25 (69.4)
Sexually active during lockdown, n (%)	28 (40)	13 (38.2)	15 (41.7)
Sexually active outside of household, n (%)	16 (82.9)	24 (88.2)	20 (79.2)
Number of partners compared to prior lockdown was:			
Fewer	52 (74.3)	28 (82.4)	24 (66.7)
Same	17 (24.3)	5 (14.7)	12 (33.3)
More	1 (1.4)	1(2.9)	-
Attitudes around COVID-19:			
Concerned about catching COVID-19, n (%)	33 (51.4)	19 (55.9)	24 (66.6)
Agree with the lockdown measures, n (%)	62 (88.6)	31 (91.1)	31 (86.1)
Wear face masks (before legally mandated), n (%)	25 (35.7)	8 (23.5)	17 (47.2)
Sexual health testing during lockdown:			
Tested quarterly for HIV/STIs, n (%)	17 (24.3)	11 (32.4)	6 (16.7)
Testing prevented due to lockdown measures, n (%)	23 (32.9)	13 (38.2)	10 (27.8)
Aware of Frisky Wales Postal Testing, n (%)	33 (47.1)	18 (52.9)	15 (41.7)

NB. Participants were subdivided into 'higher' and 'lower' risk behaviours based on the presence of one or more self-identified risk factors associated with greater odds of HIV/STI infection. These risk factors (past 12 months pre-COVID) included: diagnosis of chlamydia, gonorrhoea, syphilis, or hepatitis B &C, reports of condomless anal sex, five or more partners within an average month, or participation in chemsex (sexual intercourse under effects of mephedrone, crystal methamphetamine or GHB/GBL).
